# Role of protein arginine methyltransferase 5 over-expression in HTLV-1-driven cellular transformation and leukemia

**DOI:** 10.1186/1742-4690-12-S1-P18

**Published:** 2015-08-28

**Authors:** Amanda R Panfil, Jacob J Al-Saleem, Jesse Kwiek, Robert A Baiocchi, Patrick L Green

**Affiliations:** 1 Center for Retrovirus Research, Comprehensive Cancer Center and Solove Research Institute, USA; 2 Department of Veterinary Biosciences, Comprehensive Cancer Center and Solove Research Institute, USA; 3 Department of Microbial Infection and Immunity, Comprehensive Cancer Center and Solove Research Institute, USA; 4 Department of Molecular Virology, Immunology and Medical Genetics, Comprehensive Cancer Center and Solove Research Institute, USA; 5 The Ohio State University, Columbus, OH 43210, USA

## 

Human T-cell leukemia virus-1 (HTLV-1) is a retrovirus that infects an estimated 15-25 million people worldwide. HTLV-1 is the causative infectious agent of adult T-cell leukemia/lymphoma (ATL) and a neurodegenerative disease (HAM/TSP). While the probability of presenting any symptoms related to HTLV-1 infection is relatively low (5-10% for the lifetime of an infected individual), the disease progression and prognosis of those infected individuals who develop ATL is fatal, with a median survival range of 8-10 months. Unfortunately, ATL is highly chemotherapy resistant and while many current therapies improve ATL patient survival, the patients consistently relapse. Therefore, a need exists to develop treatments that improve ATL outcome. We have recently identified PRMT5 (protein arginine methyltransferase 5) as a potential target to modulate HTLV-1 gene expression. We find that PRMT5 levels are elevated in T-cell leukemia/lymphoma cell lines compared to freshly isolated naïve T-cells. Likewise, PRMT5 levels are elevated in total PBMCs isolated from ATL patients. However, PRMT5 RNA levels do not correlate to PRMT5 protein levels, suggesting a possible post-transcriptional method of regulation. Furthermore, we also show that PRMT5 levels are elevated during T-cell transformation by using HTLV-1 short-term immortalization assays. Utilizing shRNA vectors, we demonstrate that knockdown of endogenous PRMT5 results in decreased cellular proliferation and increased HTLV-1 viral gene expression. In addition, we observe a decrease in cell proliferation and an increase in viral gene expression when HTLV-1-infected/-transformed cells are treated with a novel small molecule inhibitor of PRMT5 (PRMT5i). Conversely, PRMT5i does not induce re-activation from HIV-1 chronically infected cells. We previously reported a protein-protein interaction between the HTLV-1 accessory protein, p30, and PRMT5. We further show PRMT5 enhances the ability of p30 to inhibit viral gene expression. In conclusion, we find PRMT5 to be a negative regulator of HTLV-1 and a potential target in HTLV-1-mediated disease.

